# Floristic Diversity and Use of Woody Plants in the Magayel and Barkaya Forests (Far North, Cameroon)

**DOI:** 10.1155/tswj/8418084

**Published:** 2026-10-23

**Authors:** Barnabas Sounlaouna, Moksia Froumsia, Taffo Junior Baudoin Wouokoue, Alphonse Diguera

**Affiliations:** ^1^ Department of Biological Sciences Faculty of Sciences University of Maroua Maroua Cameroon uni-maroua.citi.cm

**Keywords:** conservation, disturbance, Magayel and Barkaya forests, plant uses, traditional knowledge, woody diversity

## Abstract

The Magayel and Barkaya forest massifs located in the Far North Region of Cameroon are rich in biodiversity. In this study, we sought to document the floristic diversity and the use of plant resources by the local populations surrounding the Magayel and Barkaya forest massifs. A total of 12 transects (30 × 1000 m) were established in each forest massif to inventory the woody plants from May to July 2022. Data on plant uses were obtained through a survey conducted in eight villages near the forest massifs and in the nearest markets, involving 436 key informants and six focus group discussions. In total, 69 species distributed across 41 genera and 23 families were recorded in the two massifs. The Shannon diversity index varied from 3.35 to 3.43 bits and Pielou evenness index varied from 0.45 to 0.49, respectively, in Magayel and Barkaya forest massifs. The species with high value importance indices in the Magayel forest massif were *Balanites aegyptiaca* (12.74) and *Terminalia macroptera* (9.83), whereas in the Barkaya forest massif, they were *Balanites aegyptiaca* (10.80) and *Sclerocarya birrea* (10.62). Leaves, flowers, seeds, fruits, and wood are the main products exploited. The species with high uses citation frequency were *Balanites aegyptiaca* (7.74%), *Combretum fragrans* (6.45%), *Terminalia macroptera* (5.81%) in the Magayel forest massif and *Sclerocarya birrea* (4.43%), *Balanites aegyptiaca* (3.94%), *Sterculia setigera* (3.94%) in the Barkaya massif. Habitat destruction, overexploitation of natural resources, fires, and urbanization constitute the main threats to the Magayel and Barkaya forest massifs. Ensuring the sustainability of harvested products, high‐demand species should be domesticated for effective long‐term management.

## 1. Introduction

Floristic surveys involve cataloging all plant species present in a given geographic region [1]. Knowledge of the floral diversity of a particular area can reflect the totality of resources, their traditional uses, and their conservation status, which is very useful for developing conservation strategies and policies [2, 3]. Plants are considered as the most important component of natural resources upon which people and animals heavily depend. Forest areas are important for maintaining the ecological balance of nature, stabilizing the water cycle, and constitute a source of income, serve as carbon sinks, and regulate water resources [4]. Therefore, to maintain ecological balance and meet forestry needs, it is important to conduct a systematic floristic inventory and ecological survey to assess the success of natural forest conservation efforts [5]. The forests support rich biodiversity and are subject to intense anthropogenic disturbances because of the combined effects of increasing population growth and drought [6]. These diverse pressures prevent the conservation and sustainable management of these resources [7]. These ecosystems contain a wide variety of nontimber forest products. Forests play a fundamental role in the conservation of flora and fauna and in combating climate change [8–10].

Major people of the rural area directly depend on forest resources for meeting their daily requirements as well as economic well‐being [11]. The plant world provides society with a wide array of goods and services, and plant‐use practice is found to vary according to location, tradition, climatic condition, vegetation type, and rooted belief system of locals [12]. The components of biodiversity are the source of all our food and nutrition and many of our medicines, vegetables, fibers, fuels, timbers, condiments, dyes, fruits, meats, and building materials and industrial products. Hence, plant resources can be the major source of rural income and an important source of income to the government [13]. Humankind derives considerable benefits not only from the products of biodiversity but also from the services of ecological systems, such as water purification, erosion control, and pollination [14].

Beside the role played by Magayel and Barkaya forest massifs in the climate and environmental regulation, conservation of biodiversity, production of nontimber forest products for local people, we observed the reduction of their surface mainly from anthropogenic disturbances such as agriculture, production of charcoals, overgrazing and bad technic of harvesting.

Previous studies in the Far North Region of Cameroon have focused on floristic diversity [7, 15–17], medicinal plants [18], and food plants [19]. No such study was previously done in the woody plant diversity of the Magayel and Barkaya forest massifs and its importance to the local population. Most of the traditional knowledge and information about plant resources of rural areas of Magayel and Barkaya are still unknown. Therefore, it is quite important to explore more and document woody diversity and traditional knowledge about forest resources of different communities of Magayel and Barkaya forest massifs before it is completely lost. This study is aimed at carrying out a phytosociological study of woody species associated with local knowledge of derived plant products from the Magayel and Barkaya forest massifs.

## 2. Materials and Methods

### 2.1. Study Area

The study was conducted in two forest massifs (Barkaya and Magayel) located in the Kaï‐Kaï Subdivision, Mayo‐Danay Division, Far‐North Region of Cameroon. The Barkaya forest area is located in the villages of Madalam, Mbouktang, Kaï‐Kaï, and Barkaya, and the Magayel forest area is located in the villages of Sila, Djoffa, Magayel, and Manga (Figure 1). The climate is Sudano‐Sahelian type characterized by two seasons: a short rainy season (July to October) and a long dry season (November to June). The annual average rainfall varied from 700 to 800 mm. The annual average temperature is around 27.90°C [20]. The soils are clayey sandy, sandy, and hydromorphic. The landscape is punctuated by a few hills with an average altitude of 600 m.

**Figure 1:**
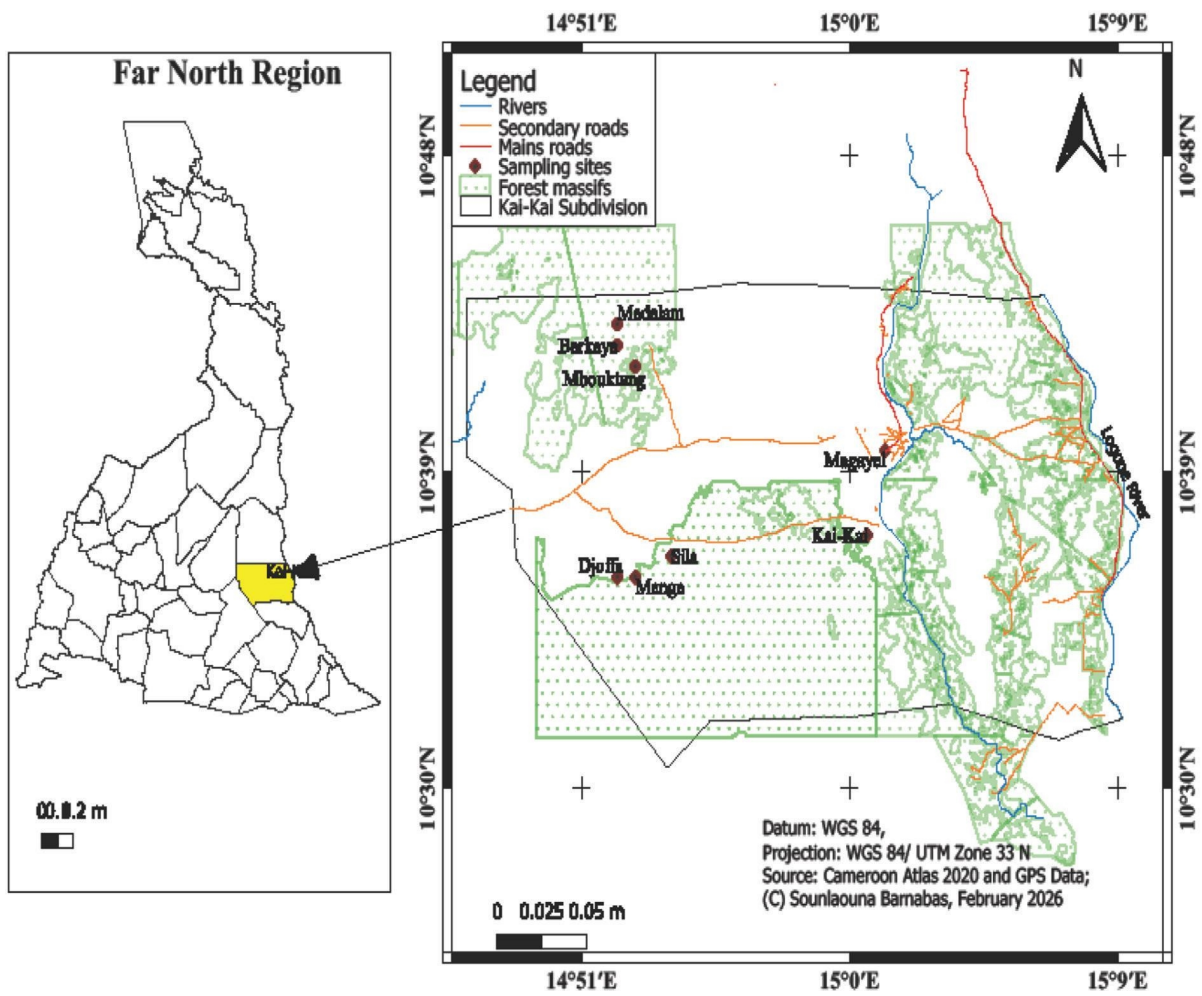
Map of the study area.

### 2.2. Floristic Data Collection

Floristic inventories were conducted during the months of June and July 2022 in the Barkaya and Magayel forest massifs. Twelve transects of 1000 × 30 m equidistant of at least 500 m were carried out in each forest massif, for a cumulative area of 36 ha per massif using Hall and Bawa methods [21]. Within each transect, all species taller than 1.5 m were identified and counted. Species were identified in situ using some books: trees, shrubs, and lianas of West African dry zones [22] and trees and shrubs of the Sahel [23]. All species were identified in the field. The final verification/validation of certain complex taxa was conducted at the National Herbarium of Cameroon (HNC). The Angiosperms Phylogeny Group classification system was used to classify the families.

### 2.3. Ethnobotanical Surveys

Information regarding plant use was collected from the local communities by implying 436 key informant (senior citizens, local healer, school teacher, and farmers) interviews and six group discussions with their prior verbal consent were conducted from March to May 2022. Informants mainly include farmers, livestock farmers, and other activities (traders, civil servants, and some retirees). The gender of the respondents was chosen randomly. Villages were chosen based on proximity and accessibility to the forest massifs. Selection of respondents was based on the age and knowledge of forest massifs. Respondents were within the age group of 15–70 years (male and female). Semistructured questionnaires were used to obtain detailed information regarding knowledge on plant resources, use‐value, harvesting methods, and conservation practices (File S1). Plant specimens and field photographs were shown during interviews and group discussions in the villages and markets. The information collected included the local name of plants and different uses.

### 2.4. Data Analysis

The species composition of the different forest massifs was described using the following parameters and formulas:

− Species richness is the total number of species in the community studied;
− Density (d) is calculated as the number of individuals per hectare;
− Basal area (Ba): Ba = *π*d2/4, where d was the diameter of individual;
− Relative density (Rd): Rd = (density of species *i*/Total density) × 100;
− Relative dominance (Rdo): Rdo = (Basal area of species *i*/Total basal area of all species) × 100;
− Relative frequency (Rf): Rf = (frequency of species *i*/Sum of frequency of all species) × 100;
− Relative diversity (Rdi): Rdi = (number of species of family *i* /Total number of species of all families) × 100;
− Importance value index (IVI): IVI = Rdo + Rf + Rd [24];
− Family importance value (FIV): FIV = Rdo + Rd + Rdi [25];
− Shannon′s diversity index (H′) was calculated from the formula
− ${\text{H}}^{\prime }=-\sum_{i=1}^{\text{s}}\text{p}\text{i}\times \log_{2}\left(\text{pi}\right)$, where pi = ni/*N*, ni was the number of individual of species *i* and *N* the total number of individuals of all species;
− Pielou equitability index (EQ): EQ = H^’^/log_2_(S)  with S as the total number of species;
− Simpson index (D): $\text{D}=1-\sum_{\text{i}=1}^{\text{s}}{Pi}^{2}$

Relative frequency of citation (Rfc) was calculated based on usage category: Rfc = (S/N ) × 100, where S being the number of people who provided a response for a given use and *N* is the total number of people surveyed.

The ethnobotanical use value was calculated using Vu (i) = ∑Sk/n, where Sk was the number of positive responses for use category k and n was the total number of positive respondents for this use category from all species studied.

## 3. Results

### 3.1. Floristic Diversity

In total, 1294 woody individuals were recorded in the Magayel forest massif belonging to 51 species, 39 genera, and 21 families, whereas 1672 woody individuals belonging to 61 species, 41 genera, and 23 families in the Barkaya forest massif (Table 1 and Table S2). The Shannon diversity index value was high (3.43 bits) in Barkaya massif, whereas the Pielou evenness index was high (0.49) in the Magayel forest massif.

**Table 1:** Taxonomic richness and diversity indices.

Forest massifs	Taxonomic groups			Diversity indices		
	Families	Genera	Species	Shannon	Pielou	Simpson
Magayel	19	38	56	3.35	0.49	0.95
Barkaya	23	41	61	3.43	0.45	0.94

#### 3.1.1. Density and Basal Area

The total density was 35.94 stems.ha^−1^ in the Magayel massif and 46.44 stems.ha^−1^ in the Barkaya forest massif. The densest species in the Barkaya massif were *Terminalia leiocarpa* (DC.) Guill. & Perr. (1.83 stems.ha^−1^), *Pseudocedrela kotschyi* (Schweinf.) Harms (1.78 stems.ha^−1^), and *Piliostigma reticulatum* (DC.) Hochst. (1.56 stems.ha^-1^), whereas in the Magayel massif, *Senegalia ataxacantha* (DC.) Kyal. & Boatwr. (5.50 stems.ha^−1^), *Balanites aegyptiaca* (L.) Del. (4.00 stems.ha^−1^), and *Guiera senegalensis* J. F. Gmel. (3.94 stems.ha^−1^) (Table 2). The total basal area was 69.69 m^2^.ha^−1^ in the Magayel forest massif and 73.07 m^2^.ha^−1^ in the Barkaya forest massif. The species *Ficus platyphylla* Del. (2.63 m^2^.ha^−1^), *Combretum glutinosum* Perr. ex DC. (2.41 m^2^.ha^−1^), and *Terminalia macroptera* Guill.& Perr. (2.25 m^2^.ha^−1^) had the highest basal areas in the Magayel massif, whereas the species *Ficus platyphylla* (2.31 m^2^.ha^−1^) and *Sclerocaya birrea* (A. Rich.) Hochst. (2.25 m^2^.ha^−1^) had the highest basal areas in the Barkaya forest massif.

**Table 2:** Density and basal area of species in forest massifs.

Forest massif							
Magayel		Barkaya		Magayel		Barkaya	
Species	Density (stem/ha)	Species	Density (stem/ha)	Species	Basal area (m^2^.ha^−1^)	Species	Basal area (m^2^.ha^−1^)
*Terminalia leiocarpa*	1.8	*Senegalia ataxacantha*	5.5	*Ficus platyphylla*	2.63	*Ficus ingens*	2.35
*Pseudocedrela kotschyi*	1.8	*Balanites aegyptiaca*	4	*Combretum glutinosum*	2.41	*Ficus platyphylla*	2.31
*Piliostigma reticulatum*	1.7	*Guiera senegalensis*	3.94	*Terminalia macroptera*	2.25	*Sclerocarya birrea*	2.25
*Mitragyna inermis*	1.6	*Sclerocarya birrea*	3.11	*Azadirachta* *Indica*	2.18	*Terminalia leiocarpa*	2.21
*Balanites aegyptiaca*	1.5	*Terminalia leiocarpa*	2.67	*Faidherbia albida*	2.17	*Sterculia setigera*	2.19
*Mimosa pigra*	1.5	*Sterculia setigera*	2.64	*Ziziphus mauritiana*	2.15	*Tamarindus indica*	2.18
*Andira inermis*	1.4	*Feretia apodanthera*	1.44	*Piliostigma thonningii*	2.15	*Vachellia sieberiana*	2.18
*Guiera senegalensis*	1.4	*Stereospermum kunthianum*	1.36	*Ficus gnaphalocarpa*	2.13	*Faidherbia albida*	2.17
*Ximenia americana*	1.3	*Combretum glutinosum*	1.28	*Terminalia leiocarpa*	2.11	*Stereospermum kunthianum*	2.15
*Steriospermum kunthianum*	1.3	*Tamarindus indica*	1.14	*Diospyros mespiliformis*	2.11	*Hexalobus monopetalus*	2.15

#### 3.1.2. Ecological Importance of Species

The most frequent species in the Magayel forest massif were *Balanites aegyptiaca* (7.74%), *Combretum fragrans* Hoffm (6.45%), *Terminalia macroptera* (5.81%), and *Piliostigma reticulatum* (3.85%), whereas in the Barkaya massif, the species with the highest frequencies were *Sclerocarya birrea* (4.43%), *Balanites aegyptiaca* (3.94%), *Sterculia setigera* Del. (3.94%), and *Terminalia leiocarpa* (3.45%) (Table 3). The most abundant species in the Magayel forest were *Terminalia leiocarpa* (5.10%), *Piliostigma reticulatum* (4.64%), and *Balanites aegyptiaca* (4.10%), whereas in the Barkaya forest, the most abundant species were *Senegalia ataxacantha* (11.84%), *Balanites aegyptiaca* (8.61%), and *Guiera senegalensis* J. F. Gmel. (8.49%). *Combretum glutinosum* (4.19%), *Ziziphus mauritiana* Lam. (3.74%), and *Diospyros mespiliformis* Hochst. ex DC. (3.67%) were the most dominant species in Magayel forest massif, whereas in the Barkaya forest massif, the most dominant species were *Sclerocarya birrea* (3.08%), *Sterculia setigera* (3.00%), *Terminalia leiocarpa* (3.02%), and *Tamarindus indica* (2.98%). The species with high value importance indices in the Magayel forest massif were *Balanites aegyptiaca* (12.74%), *Terminalia macroptera* (9.83%), *Combretum fragrans* (8.94%), and *Piliostigma reticulatum* (8.77%), whereas the species *Balanites aegyptiaca* (10.80%), *Sclerocarya birrea* (10.62%), *Sterculia setigera* (9.58%), and *Terminalia leiocarpa* (9.14%) had high importance values in the Barkaya forest massif (Table S3).

**Table 3:** Ecological importance of species in the collection units.

Forest massifs	Species	Relative frequency (%)	Relative density (%)	Relative dominance (%)	IVI
Magayel	*Balanites aegyptiaca*	7.74	1.47	3.53	12.74
	*Terminalia macroptera*	5.81	0.11	3.91	9.83
	*Combretum fragrans*	6.45	0.56	1.93	8.94
	*Piliostigma reticulatum*	3.87	1.67	3.23	8.77
	*Terminalia leiocarpa*	2.58	1.83	3.67	8.08
	*Combretum glutinosum*	2.58	1.19	4.19	7.96
	*Ziziphus mauritiana*	3.23	0.83	3.74	7.80
	*Diospyros mespiliformis*	2.58	1.22	3.67	7.47
	*Vachellia seyal*	4.52	1.00	1.86	7.38
	*Piliostigma thonningii*	3.23	0.33	3.74	7.30

Barkaya	*Balanites aegyptiaca*	3.94	4.00	2.86	10.80
	*Sclerocarya birrea*	4.43	3.11	3.08	10.62
	*Sterculia setigera*	3.94	2.64	3.00	9.58
	*Terminalia leiocarpa*	3.45	2.67	3.02	9.14
	*Senegalia ataxacantha*	3.45	5.50	0.10	9.04
	*Guiera senegalensis*	2.96	3.94	1.18	8.08
	*Combretum glutinosum*	3.45	1.28	2.90	7.63
	*Tamarindus indica*	2.96	1.14	2.98	7.08
	*Stereospermum kunthianum*	2.46	1.36	2.94	6.77
	*Hexalobus monopetalus*	2.46	1.06	2.94	6.46

#### 3.1.3. Ecological Importance of Families

The families of Fabaceae (18.92%), Combretaceae (13.46%), and Moraceae (9.62%) were the most species diverse families in the Magayel forest massif, whereas in the Barkaya forest massif, they were Fabaceae (22.58%), Combretaceae (14.52%), and Moraceae (8.06%) the most diverse families (Table 4). Fabaceae, Moraceae, Combretaceae, and Bignoniaceae were the most dominant families in the Magayel massif with 17.84%, 15.75%, 14.80%, and 9.74% relative dominance, respectively, whereas in the Barkaya massif, Fabaceae, Combretaceae, Moraceae, and Anacardiaceae were the most dominant with 24.35%, 16.08%, 13.85%, and 8.59% of relative dominance, respectively. In the Magayel forest massif, the most ecologically important families (FIV) were Fabaceae (44.34%), Combretaceae (39.60%), and Moraceae (29.03%), and in the Barkaya forest massif, families with highest importance values were Fabaceae (74.66%), Combretaceae (46.15%), and Moraceae (23.58%).

**Table 4:** Ecological importance of families.

Forest massifs	Families	Relative density (%)	Relative diversity (%)	Relative dominance (%)	FIV
Magayel	Fabaceae	15.11	11.39	17.84	44.34
	Combretaceae	11.33	13.46	14.80	39.60
	Moraceae	3.67	9.62	15.75	29.03
	Bignoniaceae	6.61	3.85	9.74	20.19
	Rubiaceae	4.22	7.69	3.48	15.39
	Fabaceae	3.50	9.62	2.17	15.01
	Meliaceae	4.28	3.85	5.55	13.67
	Rhamnaceae	3.94	3.85	5.06	12.85
	Annonaceae	2.89	3.85	1.96	8.70

Barkaya	Fabaceae	21.28	29.03	24.35	74.66
	Combretaceae	15.56	14.52	16.08	46.15
	Moraceae	1.67	8.06	13.85	23.58
	Anacardiaceae	4.61	3.23	8.59	16.43
	Annonaceae	3.89	4.84	5.69	14.42
	Rhamnaceae	3.11	4.84	4.94	12.89
	Balanitaceae	5.56	1.61	2.86	10.03
	Capparaceae	2.78	4.84	0.12	7.74

### 3.2. Plant Products Exploited in the Magayel and Barkaya Forest Massifs

The products were harvested from 53 plant species. The various plant products exploited in the Magayel and Barkaya forests were fruits, leaves, flowers, seeds, bark, roots, fodder, fuelwood, and timber (Table 5). These products are valued to varying degrees by the local populations, and their importance depends on the type of use. The different types of use were food, livestock feed, traditional medicine, fuelwood, timber, and sale to generate substantial incomes.

**Table 5:** Products exploited and their uses by the populations.

Part of plant use	Citation frequency of uses (%)				
	Foods	Folder	Medicinal	Energy	Timber
Fruits	62.60	25.64	11.76	—	—
Leaves	25.00	54.42	20.58	—	—
Flowers	97.44	2.56	—	—	—
Roots	—	—	100	—	—
Barks	—	2.56	82.35	—	—
Seeds	86.25	—	13.75	—	—
Wood	—	—	—	72.25	27.75

### 3.3. Ethnobotanical Use Value of Species

The most used species were *Tamarindus indica* L. with use value of 0.26, followed by *Balanites aegyptiaca* (0.22), *Ziziphus mauritiana* (0.21), *Sclerocarya birrea* (0.20), *Bombax costatum* Pellegr. & Vuillet (0.18), and *Azadirachta indica* A. Juss. (0.15) (Table 6).

**Table 6:** Use value of species around the forest massifs of Barkaya and Magayel.

Species	Food use	Medicinal	Fodder	Wood services	Total
*Faidherbia albida*	—	0.02	0.08	0.03	0.14
*Senegalia ataxacantha*	—	—	0.01	—	0.01
*Vachellia nilotica*	—	0.03	0.01	0.02	0.06
*Vachellia polyacantha*	—	—	0.02	0.00	0.02
*Vachellia seyal*	—	0.01	0.03	—	0.04
*Vachellia sieberiana*	—	—	0.04	0.03	0.07
*Adansonia digitata*	0.04	—	0.02	—	0.06
*Andira inermis*	0.02	—	—	0.01	0.03
*Annona senegalensis*	0.04	0.03	—	—	0.07
*Terminalia leiocarpa*	—	0.03	0.03	0.08	0.14
*Azadirachta indica*	—	0.06	—	0.09	0.15
*Balanites aegyptiaca*	0.13	0.04	0.02	0.03	0.22
*Bauhinia rufescens*	—	0.05	0.00	—	0.05
*Bombax costatum*	0.08	0.03	0.00	0.08	0.18
*Calotropis procera*	—	0.04	—	—	0.04
*Capparis sepiaria*	0.03	—	0.01	—	0.03
*Celtis integrifolia*	0.04	—	0.01	—	0.05
*Combretum aculeatum*	—	—	0.01	—	0.01
*Combretum collinum*	—	0.01	—	0.04	0.05
*Combretum fragrans*	—	—	—	0.03	0.03
*Crateva adansonii*	0.02	—	—	—	0.02
*Dalbergia melanoxylon*	—	0.01	—	—	0.01
*Dichrostachys cinerea*	—	—	0.04	0.04	0.07
*Diospyros mespiliformis*	0.03	0.03	—	0.05	0.10
*Feretia apodanthera*	—	0.00	0.02	—	0.02
*Ficus gnaphalocarpa*	0.01	—	0.03	—	0.04
*Ficus ingens*	0.01	—	0.02	—	0.03
*Ficus platyphylla*	0.01	—	0.02	0.01	0.04
*Ficus sycomorus*	0.01	0.01	0.03	0.01	0.06
*Ficus thonningii*	0.01	—	0.02	0.04	0.06
*Gardenia ternifolia*	0.00	—	0.02	0.03	0.05
*Grewia villosa*	0.00	0.02	—	—	0.02
*Guiera senegalensis*	—	0.08	—	0.05	0.14
*Hexalobus monopetalus*	0.03	0.02	0.04	—	0.09
*Hyphaene thebaica*	0.04	—	—	0.05	0.09
*Khaya senegalensis*	—	0.10	—	0.03	0.12
*Kigelia africana*	—	0.06	0.02	—	0.07
*Piliostigma thonningii*	0.02	0.02	—	0.04	0.08
*Prosopis africana*	—	0.03	0.04	0.06	0.14
*Pterocarpus erinaceus*	—	—	0.03	—	0.03
*Sclerocarya birrea*	0.08	0.04	0.04	0.03	0.20
*Senna singueana*	—	—	0.02	—	0.02
*Sterculia setigera*	—	—	0.05	—	0.05
*Stereospermum kunthianum*	—	0.05	0.02	0.01	0.08
*Strychnos spinosa*	0.03	0.00	0.04	—	0.07
*Swartzia madagascariensis*	—	0.02	0.03	—	0.05
*Tamarindus indica*	0.12	0.05	0.03	0.06	0.26
*Terminalia macroptera*	—	0.04	—	—	0.04
*Vitex doniana*	0.05	—	0.04	—	0.09
*Ximenia americana*	0.05	0.02	—	—	0.07
*Ziziphus mauritiana*	0.05	0.03	0.07	0.05	0.21
*Ziziphus mucronata*	—	0.01	0.02	—	0.03
*Ziziphus spina-christi*	0.03	—	0.04	0.01	0.08

#### 3.3.1. Food Use of Plant Products

A total of 24 woody plant species were used in the diet of local households living around the Magayel and Barkaya forests. Twenty‐two (22) species were used by local populations for their fruits, with *Ziziphus mauritiana* (75.35%), *Balanites aegyptiaca* (81.16%), *Tamarindus indica* (92.27%), *Hyphaene thebaica* (L.) Mart. (69.89%), and *Hexalobus monopetalus* (A. Rich.) Engl. & Diels (81.84%) as the most widely used species by local populations for their seeds, which were eaten raw, cooked, or processed (Figure 2 and Table S5). This was the case for *Balanites aegyptiaca* seeds, which were used by 53.73% of the population for consumption after cooking, like peanuts, or after cold extraction of *B. aegyptiaca* oil (Figure 3).

**Figure 2:**
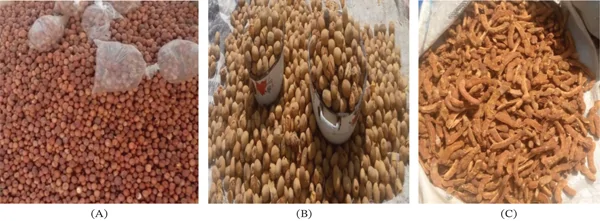
Fruits of some of the most popular species: (A) fruits of *Ziziphus mauritiana*, (B) fruits of *Balanites aegyptiaca*, and (C) fruits of *Tamarindus indica.*

**Figure 3:**
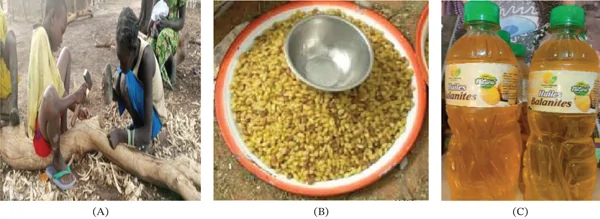
Production process of *Balanites aegyptiaca* oil. (A) Production of (B) seeds and (C) oil.

#### 3.3.2. Medicinal Uses of Exploited Products

Thirty‐four (34) species were used by these populations to treat various illnesses (Table S4). The most commonly used species were *Azadirachta indica* (58.43%), *Khaya senegalensis* (Desr.) A.Juss. (50.43%), *Vachellia nilotica* (L.) P.J.H.Hurter & Mabb. (50.29%), *Bauhinia rufescens* Lam. (42.26%), and *Tamarindus indica* (24.82%). The most commonly used plant parts were roots (100%), bark (82.35%), leaves (20.58%), fruits (11.76%), and seeds (13.75%) (Table S6).

#### 3.3.3. Fodder Use

Twenty (20) species were used for livestock feed around the forest areas (Table S7). The most used were *Ziziphus mauritiana* (37.94%), *Sterculia setigera* (36.53%), *Ficus platyphylla* (35.65%), *Faidherbia albida* (Delile) A. Chev. (34.35%), and *Tamarindus indica* (20.82%). The use of species for fodder occurs more often during the dry season when grasses become dry and increasingly scarce. The choice of these species was based on the palatable quality appreciated by the animals.

#### 3.3.4. Use for Firewood

Fifty‐four (54) species were used by the populations around the Magayel and Barkaya forest massifs for firewood (Table S8). The most used species were *Terminalia leiocarpa* (36.88%), *Balanites aegyptiaca* (32.62%), *Prosopis africana* (Guill. & Perr.) (31.33%), and *Vachellia seyal* (Delile) P.J.H.Hurter (18.36%). These species were chosen by the populations based on their characteristics: hardwood, long burning, and low ash production.

#### 3.3.5. Use of Wood for Charcoal Production

The species used by the populations surrounding the Magayel and Barkaya forest massifs for charcoal production were those with good charcoal yield, hardwood, and low ash production (Figure 4). A total of 20 species were used for charcoal production, which include *Balanites aegyptiaca* (15.00%), *Terminalia leiocarpa* (8.75%), *Prosopis africana* (7.92%), *Faidherbia albida* (6.67%), *Stereospermum kunthianum* Cham. (5.42%), *Vachellia sieberiana* (DC.) Kyal. & Boatwr. (5.42%), and *Vachellia seyal* (5.00%).

**Figure 4:**
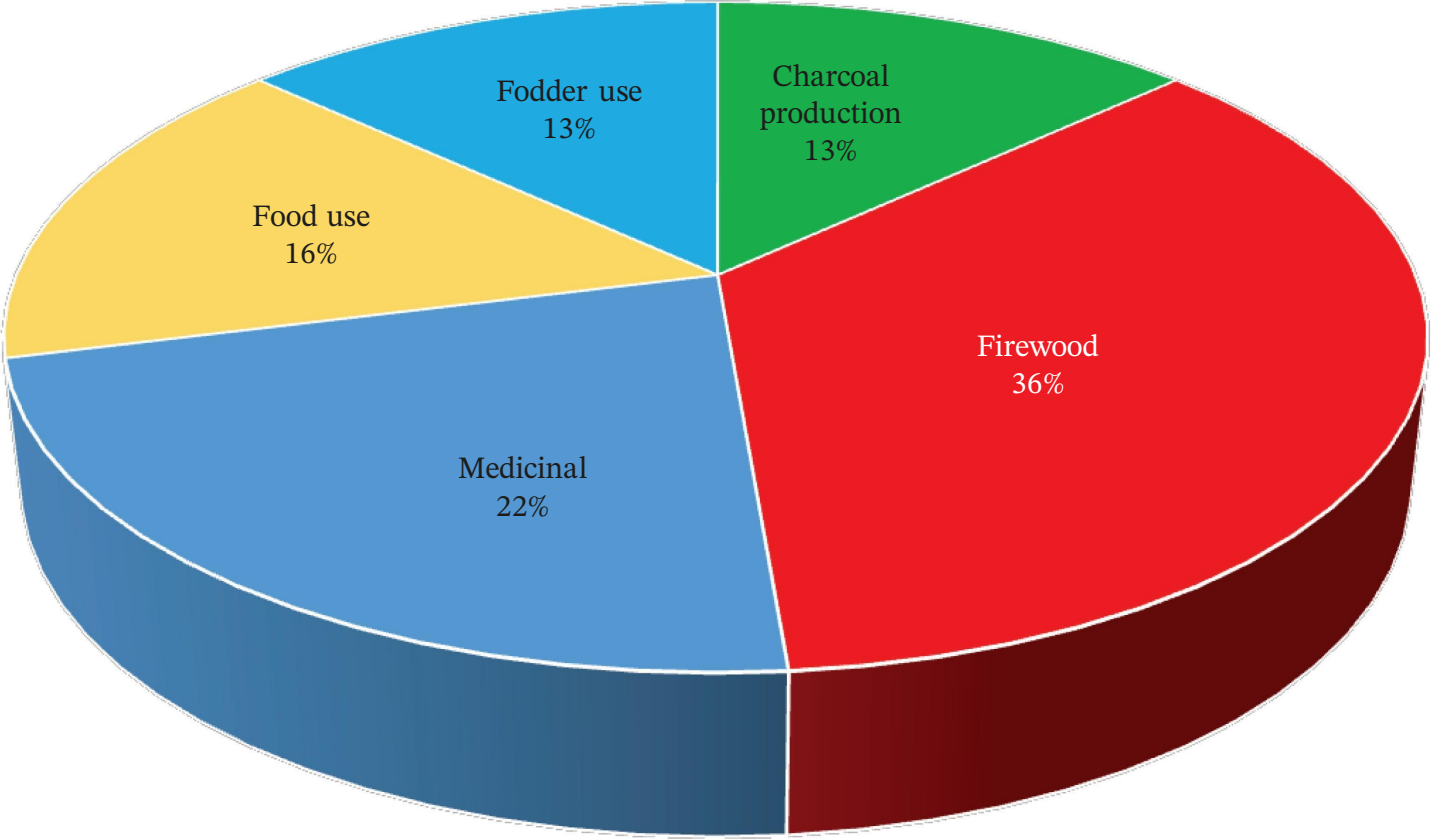
Different uses of plants in Magayel and Barkaya forest massifs.

#### 3.3.6. Marketing and Income Generation

The quality of nontimber forest products collected for sale provides substantial income to the household economy in the area (Figure 5). A total annual income of 27,833,499 XAF was recorded with 62,971 XAF per farmer. Leaves contribute to 2,708,333 XAF, flowers to 61,800 XAF, fruits to 2,437,500 XAF, and seeds to 3,514,000 XAF. Combining these incomes with agricultural incomes allowed farmers to cope with the food deficit and ensure their health care as well as the schooling of children. The harvesting of these products is done seasonally by rural farmers and is mainly done by women and children. This strong contribution of the marketing of these products in the household economy leads to a rush of populations on resources.

**Figure 5:**
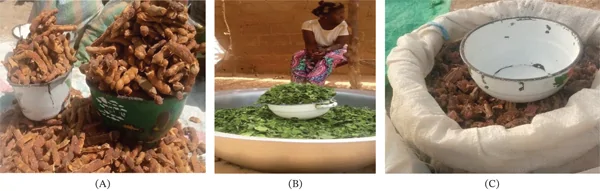
Sale of (A) *Tamarindus indica* fruits, (B) *Balanites aegyptiaca* leaves, and (C) *Bombax costatum* flowers.

Regarding firewood, the quantity sold was estimated at 1982.88 t for an estimated annual income of 17,347,500 XAF. The transport of wood to the market is done mainly by women who carry it either on their heads or on the backs of donkeys and also by men who transport it on motorcycles. The cost of transport varies depending on the quantity and the period. The populations of this area use wood (99%) as an energy source, which leads to deforestation.

## 4. Discussion

The results showed that the two forest massifs were rich in woody plant species. The taxonomic richness was lower than 78 woody species belonging to 53 genera and 24 families found in the Goulourgou‐Wina forest massif [6], and higher than the 44 woody species found in the Pette forest massif by [26] and 42 woody species obtained in the Diamare woodlands, all in the Far North Region of Cameroon [27]. Such variation reflects differences in disturbance intensity across sites, particularly in relation to wood harvesting and grazing. The Shannon diversity indices of 3.35–3.43 bits suggest that the forest still maintains a relatively stable community structure despite ongoing human pressures. This finding was similar to some studies in other forest massifs in the Far North Cameroon [6, 26].

The dominance of *Combretum glutinosum* and *Ziziphus mauritiana* could be explained by the high number of individuals of these species. The dominance of these species indicates strong adaptation to disturbances but also raises concerns about the declining representation of less competitive taxa. The total density was lower compared with 59.7 stems.ha^−1^ found in noncultivated plains of Mourtourwa [15]. This could be explained by the intensity of human pressures on the vegetation. Species with high IVI were the most dominant, the most frequent, and the densest. Those with low IVI are particularly vulnerable to local extinction if anthropogenic pressures persist. These results indicated the need for targeted conservation of underrepresented taxa through domestication or enrichment planting in both forests. [6] showed that protecting this component of floristic diversity is critical not only for maintaining ecological balance but also for safeguarding the ecosystem services that local communities depend upon.

Fabaceae and Combretaceae contributed most to overall richness and dominance, consistent with patterns observed elsewhere in northern Cameroon and neighboring Chad [28, 29]. The ecological success of Fabaceae can be attributed to traits such as efficient nitrogen fixation and resilience to environmental stress [30]. This result was similar to those of [29] who found that these families were the most abundant and diverse in the Mourtourwa reserve and surroundings.

The locals people of Magayel and Barkaya forests depend upon the forest for their primary healthcare, fodder, food, fuelwood, timber, trade, and other miscellaneous purposes, and the plant products harvested were leaves, flowers, fruits, roots, bark, and wood. The number of plants harvested was higher than 48 plant species found by [31] in Logone Plain Far North Cameroon. People preferred to collect species with multiple uses, and the population of those plants was more disturbed because of overharvesting and developmental activities. The number of food plants was similar to 24 edible fruit tree species obtained [29] in the reserve of Moutourwa and its surroundings but lower than 38 wild edible fruit species found [32] in Maroua and the 36 fruit species found [31] in Logone valley (Far North Cameroon).

The number of plants used in traditional pharmacopeia was lower than those of [32] who found 50 plants used by local population in Mount Mandara in Cameroon. The interest in roots, barks, leaves, fruits, and seeds can be explained by the fact that these plant organs are the excellent seat of biosynthesis and storage of secondary metabolites which are responsible for the plants pharmacobiological properties. Results showing high frequency use of roots and barks were also obtained in Tomboronkoto, Kédougou (Senegal) [33]. Uncontrolled use of certain organs such as roots and barks could cause damage to the plants and even its ecosystem [29]. So, good harvesting techniques must be used to preserve it for us and for future generations.

The energy source for cooking by the local population was wood and charcoal, which remain the only easily accessible energy. These results were similar to those of [34] who showed that wood energy was the main energy source of households in some cities in the Sahelian zone of Africa. Significant quantities of fuelwood (94.5 t/year of wood) harvested around and within the Magayel and Barkaya forest massifs and sold in the city of Maroua were from Kaï‐Kaï [35]. Despite their multiple uses, some wild food trees were slaughtered for firewood or to produce charcoal. Plants used for producing charcoal were fewer than 24 species obtained [32]. The choice of species to produce charcoal can depend on the availability of plant material and quality of the wood. The high exploitation of woody plants for charcoal and firewood causes desertification in Sahelian area which begins with deforestation. This degradation leads to irreversible extinction of biodiversity and the decline of the reproductive potential of woody resources [36]. Inappropriate and overexploitation of plant species may become harmful if they exceed sustainable regeneration. Wild species that are exploited to produce charcoal should be integrated into reforestation plans, especially multipurpose species that are already considered to be endangered locally. This harvesting would lead to a reduction in vegetation cover and threaten sparse species if sustainable protection and preservation measures are not implemented. The number of fodder plants was lower than the 31 fodder plants found [37] in the Sahelian zone of Cameroon.

The diversity of products, the size of the quantity harvested, and their availability all year round allows farmers to fill the deficit in agricultural production and ensure daily food security for the family. The average annual income of 62,971 XAF per farmer was higher than 55,000 XAF/operator estimated income per farmer around the exploitation of *Borassus aethiopum* reported in Niger [38] and the income of 11,618 XAF/inhabitant from forestry activities by households in the center‐west of Burkina Faso [34].

## 5. Conclusion

The Magayel and Barkaya forest massifs host high plant diversity, confirming their importance as a biodiversity reservoir in the Sudano‐Sahelian zone. These forests showed moderate diversity indices and the dominance of Fabaceae and Combretaceae families. The local livelihood system depends heavily on traditions and values that are deeply rooted. Locals gave priority to those wild woody plant species that provide them with multitude of benefits as *Balanites aegyptiaca*, *Tamarindus indica*, *Bombax costatum*, and *Ziziphus mauritiana*. Habitat destruction, overharvesting of natural resources, wildfire, and unmanaged road construction are the major threats to the natural forest.

## Funding

No funding was received for this manuscript.

## Disclosure

The final manuscript was read and approved by all authors.

## Ethics Statement

The study involves human participants sharing traditional ecological knowledge about plant uses for academic purpose. Individual informed consent: Before interviewing any local community member, this key information has been disclosed. Identification: name and affiliation of the researcher. Purpose: Plant food survey is being conducted to preserve local knowledge of useful plants. Voluntary participation: Participation is entirely voluntary and that participants can skip questions or withdraw at any time. Data handling and anonymity: Personal identity and traditional knowledge will be anonymized. Benefit sharing: Copy of final report will be sent to the community.

## Consent

The authors have nothing to report.

## Conflicts of Interest

The authors declare no conflicts of interest.

## Supporting information


**Supporting Information** Additional supporting information can be found online in the Supporting Information section. File S1: Ethnobotanical survey. Table S1: Species checklist of two forest massifs. Table S2: Ecological importance value of all species. Table S3: List of medicinal plants including the parts used, methods of preparation, route of administration, and treated diseases. Table S4: Citation frequencies of food species in the two forest massifs. Table S5: Citation frequencies of medicinal species in the two forest massifs. Table S6: Citation frequencies of fodder species in the two forest massifs. Table S7: Citation frequencies of firewood species in the two forest massifs.

## Data Availability

The data that support the findings of this study are available from the corresponding author upon reasonable request.
