# Supplementary material for: Floristic Diversity and Use of Woody Plants in the Magayel and Barkaya Forests (Far North, Cameroon)
Source: ScientificWorldJournal. 2026 Oct 23;2026:8418084. doi: 10.1155/tswj/8418084 (PMC13691628; doi:10.1155/tswj/8418084)
Supplement: Supplementary file 1 [file TSWJ-2026-8418084-s001.docx]

**Supplementary material**

Supplementary File S1 : Ethnobotanical survey

I. **Respondent identification**

Name and first name………………..Ethnic group…………….Village……… Age……. Sex : F □ M □	Level of education : …………Number of people in the household………..Marital status : Single □ Married □ Monogamous	□ Widower □ Widow □ Polygamous □ Main activity…………………………..….Secondary activity……………………………………………………………………………………………………………….

II. **Knowledge of forest areas**

1. Do you know this forest area ?................................................................................................................................

2. Can you delineate this forest area ?.........................................................................................................................

3.What are the main characteristics of this forest area ?.............................................................................................

4. What is the importance of this forest area to you ?.................................................................................................

5. What are the main human activities in these forest massifs ?.................................................................................

6. Who manages this forest area?...........................................Is it well managed ?........................Do you contribute to its management ?………………..Otherwise, why ?.............................................................................

7. What improvements need to be made for its proper management ?.......................................................................

8. Do you harvest products from these forest areas ?.............................................if so, provide the types…………

9. Which ones are more important to you ?................................................................................................................

**III. Identification of the main non timber forest products**

1. What are the different non-wood forest products you harvest in the forest area ?

| Common name | Scientific name | Products | Main uses | Harvesting periods | Harvesting method | Availability |
| --- | --- | --- | --- | --- | --- | --- |
|  |  |  |  |  |  |  |
|  |  |  |  |  |  |  |
|  |  |  |  |  |  |  |
|  |  |  |  |  |  |  |
|  |  |  |  |  |  |  |
|  |  |  |  |  |  |  |
|  |  |  |  |  |  |  |

2. Which species are the most important to you ?

| Common name | Scientific name | Products handled | Availability | Regeneration | Use |
| --- | --- | --- | --- | --- | --- |
|  |  |  |  |  |  |
|  |  |  |  |  |  |
|  |  |  |  |  |  |
|  |  |  |  |  |  |

3. Which ones have been introduced into your operating systems ?...........................................................................

**IV. Importance of non-timber forest products (NTFPs) for the harvester and the household economy.**
1. What are the main products you harvest?…………………………………………………………………..…….
2. At what time of year does this harvesting take place?............................................................................................
3. Do you collect NTFPs for sale? Yes □ No □
4. If yes, please provide the following details. Which ones?……………………………………………………..…
……………………………………………………………………………………………………………….………
5. What do you do with the income generated from the sale of these products?…………………………………....
6. In your opinion, what proportion of households harvest NTFPs?..........................................................................
7. In your opinion, what proportion of households in the village sell NTFPs?..........................................................
8. Do you process NTFPs? Yes □ No □. If yes, what are the processed products?....................................................
9. What types of processing are involved?..................................................................................................................
10. Why do you carry out this processing?.................................................................................................................
…………………………………………………………………………………………………………………….....
**V. Conservation of natural resources and NTFP**
1. In your opinion, what has been the trend regarding the diversity of natural resources in the reserve over the past ten years? (Please comment and provide reasons)…........................................................................................... 2. Do you have natural resources available to combat inequalities?...........................................................................
3. Do you think you can better encourage nations to conserve their resources?.....................................................
4. Is there a technique for environmental monitoring?...............................................................................................
5. Among the various non-timber forest products (NTFPs), do any appear to be overexploited?..............................
6. What might be the future consequences of this overexploitation?..........................................................................
7. The staff has methods available to protect the reserve……………………………………………………………
8. Participate in the latest wine improvement methods……………………………………………………………...

**VI. Product Recalls**
1. Here are the main products you have introduced to the natural environment:

| Especies | Main uses | Others uses | Harvesting method | Harvested period | Sall period |
| --- | --- | --- | --- | --- | --- |
|  |  |  |  |  |  |
|  |  |  |  |  |  |
|  |  |  |  |  |  |
|  |  |  |  |  |  |
|  |  |  |  |  |  |
|  |  |  |  |  |  |
|  |  |  |  |  |  |
|  |  |  |  |  |  |

2. For each of these products, indicate the quantities harvested (50 kg bags, [100](tel:100) kg bags, basins, buckets, cartons, etc.)…………………………………………………………………………………………………………………..
3. Do you seek authorization from administrative authorities?..................................................................................
4. Do you pay taxes? yes □   no □  if yes, list them and state the amount………………………………………......
…………………………………………………………………………………………………………………….…
5. What is the usual unit of sale?.................................................................................................................................
6. Place of sale: home □   school □  roadside □ market □ other (specify)
7. What are the main sales markets?...........................................................................................................................
8. How long have you been harvesting and selling these products?...........................................................................
9. Who are your main customers?...............................................................................................................................
10. Method of sale: retail □ wholesale □ semi-wholesale □ other (specify) □
11. How are the products stored?................................................................................................................................
12. How many times a month do you sell these products?.........................................................................................
13. What is your monthly income from this?..............................................................................................................
14. Among these products, indicate which ones are sold outside the country……………………………………………………………………………………………………………….16. Are these manufactured products consumed exclusively by your household?.....................................................
17. If not, what quantities do you sell on average and at what price?
18. Do you find these fruits in sufficient quantities in the bush? Yes □ No □
19. Which ones are most sought after on the market?

VII. Firewood
1. What are the main species harvested for firewood and charcoal?

| Espèce | Bois de feu | Charbon | Qualité de produits | Disponibilité |  |  |
| --- | --- | --- | --- | --- | --- | --- |
|  |  |  |  |  |  |  |
|  |  |  |  |  |  |  |
|  |  |  |  |  |  |  |
|  |  |  |  |  |  |  |

2. What are the different cutting methods used?.........................................................................................................
3. What is the purpose for which you harvest these different species?.......................................................................
4. What is the condition of the species you harvest?...................................................................................................
5. If you harvest for your household, what quantity do you use per month?..............................................................
6. What quantity do you sell per month? 6. Who are the buyers of the wood?……………………………………..
7. What are the main markets for selling the wood? ………………………………………………………………..
8. What is the distance traveled for cutting wood?………………………………………………………………….
9. What is the most commonly used means of transport?...........................................................................................
10. What difficulties are encountered in carrying out this activity?...........................................................................
11. Do you pay taxes? If so, how much?.....................................................................................................................
12. In your opinion, is there still sufficient wood available in the bush?....................................................................
13. How long have you been engaged in this activity?...............................................................................................
14. What is your monthly income from selling wood?...............................................................................................

15. What do you propose for the long-term sustainability of this activity?................................................................

VIII. Information on the value chain and market product prices
1. In which role are you most involved?
Gatherer □ Reseller □ Wholesaler □ Retailer □ Consumer □ Other………………..…………………………….…
2. What is the role of each of these different actors in the value chain?.....................................................................
…………………………………………………………………………………………………………………….…
3. How long have you been involved in the sale of NWFPs (Non-Wood Forest Products)?.....................................
4. What proportion of your monthly cash income is derived from the NWFP trade?................................................
5. What are the main NWFPs you trade? ………………………………………………………………………..….
6. From whom and where do you source these products? ………………………………………………….……….
7. Who are the main actors involved in the marketing of NWFPs?............................................................................
8. Among these actors in the value chain, who makes the most profit and why?.......................................................
9. What factors influence product prices on the market?............................................................................................
10. Are the products perishable?.................................................................................................................................
11. What comments or suggestions do you have regarding the value chain?............................................................

Supplementary Table S1: Species checklist of two forest massifs

| Families | Magayel species | Barkaya species |
| --- | --- | --- |
| Anacardiaceae | *Lannea fruticose*  (Hochst. ex A. Rich.) | *Sclerocarya birrea* (A. Rich.) Hochst. |
|  | *Sclerocarya birrea* (A. Rich.) Hochst. | *Lannea velutina* A. Rich. |
|  |  | *Lannea fruticose* (Hochst. ex A. Rich.) |
| Annonaceae | *Annona senegalensis* Pers. | *Hexalobus monopetalus* (A. Rich.) Engl. & Diels |
|  |  | *Annona senegalensis* Pers. |
| Arecaceae | *Hyphaene thebaica* (L.) | *Hyphaene thebaica* (L.) |
| Asclepiadaceae | *Calotropis procera* (Ait.) Ait. F | *Calotropis procera* (Ait.) Ait. f. |
| Balanitaceae | *Balanites aegyptiaca* (L.) Del | *Balanites aegyptiaca* (L.) Del. |
| Bignoniaceae | *Kigelia Africana* (Lam.) Benth. | *Stereospermum kunthianum* Cham. |
|  | *Stereospermum kunthianum* Cham. |  |
| Bombacaceae |  | *Bombax costatum* Pellegr. & Vuillet |
| Fabaceae | *Cassia sieberiana* DC. | *Bauhinia rufescens* Lam. |
|  | *Detarium microcarpum* Guill. & Perr. | *Piliostigma reticulatum* (DC.) Hochst. |
|  | *Piliostigma reticulatum* (DC.) Hochst. | *Tamarindus indica* L. |
|  | *Piliostigma thonningii* (Schumach.) | *Piliostigma thonningii* (Schumach.) |
|  | *Cassia singueana* (Del.) Lock | *Swartzia madagascariensis* Desv. |
|  | *Tamarindus indica* L. | *Senegalia ataxacantha* DC. |
|  | *Andira inermis* D.C. | *Dichrostachys cinerea* (L.) Wight & Arn. |
|  | *Mimosa pigra* L. | *Vachellia sieberiana* DC. |
|  | *Prosopis Africana* (Guill. & Perr.) | *Acacia Senegal* (L.) Willd |
|  | *Vachellia seyal* Del. | *Vachellia seyal* Del. |
|  | *Vachellia sieberiana* DC. | *Vachellia nilotica* (L.) |
|  | *Dichrostachys cinerea* (L.) Wight & Arn. | *Faidherbia albida* Del. |
|  |  | *Senegalia polyacantha* Willd. |
|  |  | *Parkia biglobosa* (Jacq.) R.Br.ex *G. Don* |
|  |  | *Prosopis Africana* (Guill. & Perr.) |
|  |  | *Albizia lebbeck* (L.) |
| Capparaceae |  | *Capparis sepiaria* L. |
|  | *Capparis sepiaria* L. | *Capparis corimbosa* Forssk. |
|  | *Cadaba farinose* Forssk | *Cadaba farinose* Forssk. |
| Celastraceae |  | *Maytenus senegalensis* (Lam.) Exell |
| Combretaceae | *Terminalia leiocarpa* (DC.) Guill.& Perr. | *Terminalia leiocarpa* (DC.) Guill. & Guill. & Perr. |
|  | *Combretum aculeatum* Vent. | *Terminalia macroptera* Guill.& Perr. |
|  | *Combretum fragrans* Hoffm | *Terminalia ternifolia* Schumach*.* & Thonn |
|  | *Combretum glutinosum* Perr. ex DC. | *Combretum glutinosum* Perr. ex DC. |
|  | *Guiera senegalensis* J. F. Gmel. | *Combretum aculeatum* Vent. |
|  | *Terminalia avicennioides* Guill. & Perr. | *Guiera senegalensis* J. F. Gmel. |
|  | *Terminalia macroptera* Guill.& Perr. | *Combretum fragrans* Hoffm |
|  |  | *Terminalia avicennioides* Guill. & Perr. |
|  |  | *Combretum molle* R.Br. |
| Ebenaceae | *Diospyros mespiliformis* Hochst. ex DC. | *Diospyros mespiliformis* Hochst. ex DC. |
| Euphorbiaceae | *Fluggea virosa* (Roxb. Ex Willd.) Voigt | *Flueggea virosa* (Roxb. Ex Willd.) |
| Loganiaceae |  | *Strychnos spinose* Lam. |
| Meliaceae | *Azadirachta indica* A. Juss. | *Azadirachta indica* A. Juss. |
|  | *Pseudocedrela kotschyi* (Schweinf.) Harms |  |
| Moraceae | *Ficus gnaphalocarpa* (Miq.) | *Ficus sycomorus* (Miq.) C.C. Berg |
|  | *Ficus ingens* (Miq.) Miq. | *Ficus ingens* (Miq.) Miq. |
|  | *Ficus platyphylla* Del. | *Ficus thonningii* Blume |
|  | *Ficus sycomorus* (Miq.) C.C. Berg | *Ficus gnaphalocarpa* (Miq.) |
|  | *Ficus thonningii* Blume | *Ficus platyphylla* Del. |
| Olacaceae | *Ximenia Americana* L. | *Ximenia Americana* L. |
| Papilionanaceae | *Dalbergia melanoxylon* Guill. & Perr. | *Pterocarpus erinaceus* Poir. |
|  | *Pterocarpus erinaceus* Poir. | *Dalbergia melanoxylon* Guill. & Perr. |
| *Rhamnaceae* | *Ziziphus mauritiana* Lam. | *Ziziphus mauritiana* Lam. |
|  | *Ziziphus mucronata* Willd. | *Ziziphus mucronata* Willd. |
|  |  | *Ziziphus spina-christii* L. |
| Rubiaceae | *Feretia apodanthera* Del. | *Gardenia ternifolia* Schumach. & Thonn. |
|  | *Gardenia ternifolia* Schumach. & Thonn. | *Feretia apodanthera* Del. |
|  | *Mitragyna inermis* (Willd.) K. Schum. |  |
| Sterculiaceae | *Sterculia setigera* Del. | *Sterculia setigera* Del. |
| Tiliaceae | *Grewia barteri* Burret | *Grewia villosa* Willd. |
|  | *Grewia flavescens* Juss. | *Grewia flavescens* Juss. |
|  | *Grewia villosa* Willd. |  |
| *Ulmaceae* | *Celtis integrifolia* Lam. | *Celtis integrifolia* Lam. |
| *Verbenaceae* |  | *Vitex doniana* Sweet |

Supplementary Table S2: Ecological importance value of all species

| Magayel forest massif | | | | |
| --- | --- | --- | --- | --- |
| Species | Relative frequency (%) | Relative density (%) | Relative dominance (%) | IVI |
| *Balanites aegyptiaca* | 7.74 | 1.47 | 3.53 | 12.74 |
| *Terminalia macroptera* | 5.81 | 0.11 | 3.91 | 9.83 |
| *Combretum fragrans* | 6.45 | 0.56 | 1.93 | 8.94 |
| *Piliostigma reticulatum* | 3.87 | 1.67 | 3.23 | 8.77 |
| *Terminalia leiocarpa* | 2.58 | 1.83 | 3.67 | 8.08 |
| *Combretum glutinosum* | 2.58 | 1.19 | 4.19 | 7.96 |
| *Ziziphus mauritiana* | 3.23 | 0.83 | 3.74 | 7.80 |
| *Diospyros mespiliformis* | 2.58 | 1.22 | 3.67 | 7.47 |
| *Vachellia seyal* | 4.52 | 1.00 | 1.86 | 7.38 |
| *Piliostigma thonningii* | 3.23 | 0.33 | 3.74 | 7.30 |
| *Annona senegalensis* | 4.52 | 0.56 | 1.96 | 7.04 |
| *Andira inermis* | 1.94 | 1.44 | 3.62 | 7.00 |
| *Faidherbia albida* | 2.58 | 0.61 | 3.77 | 6.96 |
| *Steriospermum kunthianum* | 1.94 | 1.25 | 3.46 | 6.65 |
| *Ficus platyphylla* | 1.29 | 0.75 | 4.57 | 6.61 |
| *Ximenia americana* | 3.23 | 1.28 | 2.07 | 6.57 |
| *Vachellia sieberiana* | 2.58 | 0.44 | 3.53 | 6.55 |
| *Ficus thonningii* | 2.58 | 0.53 | 3.34 | 6.45 |
| *Ficus ingens* | 4.52 | 0.14 | 1.77 | 6.43 |
| *Mitragyna inermis* | 1.29 | 1.56 | 3.37 | 6.22 |
| *Azadirachta indica* | 1.94 | 0.36 | 3.79 | 6.09 |
| *Ficus gnaphalocarpa* | 1.94 | 0.19 | 3.70 | 5.83 |
| *Sterculia setigera* | 1.94 | 0.06 | 3.56 | 5.56 |
| *Kigelia africana* | 1.94 | 0.69 | 2.66 | 5.29 |
| *Pseudocedrela kotschyi* | 1.29 | 1.78 | 1.76 | 4.82 |
| *Hyphaene thebaica* | 1.94 | 0.19 | 2.68 | 4.81 |
| *Celtis integrifolia* | 2.58 | 0.22 | 1.89 | 4.70 |
| *Ficus sycomorus* | 1.94 | 0.25 | 2.36 | 4.55 |
| *Detarium microcarpum* | 1.94 | 0.14 | 1.81 | 3.88 |
| *Sclerocarya birrea* | 1.94 | 0.17 | 1.76 | 3.86 |
| *Prosopis africana* | 1.29 | 0.67 | 1.77 | 3.73 |
| *Lannea fructicosa* | 1.94 | 0.78 | 0.97 | 3.69 |
| *Tamarindus indica* | 1.29 | 0.78 | 1.50 | 3.56 |
| *Ziziphus mucronata* | 0.65 | 1.14 | 1.32 | 3.11 |
| *Dichrostachys cinerea* | 1.94 | 1.03 | 0.07 | 3.03 |
| *Mimosa pigra* | 1.29 | 1.47 | 0.07 | 2.83 |
| *Cadaba farinosa* | 1.94 | 0.42 | 0.43 | 2.79 |
| *Terminalia avicennioides* | 0.65 | 0.06 | 1.84 | 2.54 |
| *Dalbergia melanoxylon* | 1.29 | 1.03 | 0.02 | 2.34 |
| *Guiera senegalensis* | 0.65 | 1.36 | 0.24 | 2.25 |
| *Senna singueana* | 1.29 | 0.64 | 0.07 | 2.00 |
| *Capparis sepiaria* | 1.29 | 0.64 | 0.05 | 1.98 |
| *Combretum aculeatum* | 1.29 | 0.56 | 0.10 | 1.95 |
| *Gardenia ternifolia* | 1.29 | 0.47 | 0.05 | 1.81 |
| *Fluggea virosa* | 0.65 | 0.86 | 0.02 | 1.52 |
| *Grewia villosa* | 1.29 | 0.19 | 0.02 | 1.50 |
| *Calotropis procera* | 1.29 | 0.08 | 0.07 | 1.44 |
| *Pterocarpus erinaceus* | 0.65 | 0.03 | 0.38 | 1.06 |
| *Grewia barteri* | 0.65 | 0.39 | 0.02 | 1.05 |
| *Grewia flavescens* | 0.65 | 0.36 | 0.02 | 1.02 |
| *Feretia apodanthera* | 0.65 | 0.17 | 0.05 | 0.86 |

| Barkaya forest massif | | | | |
| --- | --- | --- | --- | --- |
| Species | Relative frequency (%) | Relative density (%) | Relative dominance (%) | IVI |
| *Balanites aegyptiaca* | 3.94 | 4.00 | 2.86 | 10.80 |
| *Sclerocarya birrea* | 4.43 | 3.11 | 3.08 | 10.62 |
| *Sterculia setigera* | 3.94 | 2.64 | 3.00 | 9.58 |
| *Terminalia leiocarpa* | 3.45 | 2.67 | 3.02 | 9.14 |
| *Senegalia ataxacantha* | 3.45 | 5.50 | 0.10 | 9.04 |
| *Guiera senegalensis* | 2.96 | 3.94 | 1.18 | 8.08 |
| *Combretum glutinosum* | 3.45 | 1.28 | 2.90 | 7.63 |
| *Tamarindus indica* | 2.96 | 1.14 | 2.98 | 7.08 |
| *Steriospermum kunthianum* | 2.46 | 1.36 | 2.94 | 6.77 |
| *Hexalobus monopetalus* | 2.46 | 1.06 | 2.94 | 6.46 |
| *Acacia senegal* | 2.46 | 0.81 | 2.92 | 6.18 |
| *Vachellia seyal* | 1.97 | 1.06 | 2.89 | 5.91 |
| *Vachellia nilotica* | 1.97 | 0.81 | 2.78 | 5.55 |
| *Diospyros mespiliformis* | 1.97 | 0.64 | 2.92 | 5.52 |
| *Faidherbia albida* | 1.97 | 0.42 | 2.97 | 5.36 |
| *Terminalia ternifolia* | 1.48 | 0.44 | 2.89 | 4.81 |
| *Vachellia sieberiana* | 1.48 | 0.31 | 2.98 | 4.77 |
| *Annona senegalensis* | 1.48 | 0.53 | 2.75 | 4.76 |
| *Piliostigma reticulatum* | 1.48 | 0.47 | 2.81 | 4.76 |
| *Ficus thonningii* | 1.48 | 0.33 | 2.93 | 4.74 |
| *Bombax costatum* | 2.46 | 0.64 | 1.52 | 4.62 |
| *Ficus platyphylla* | 0.99 | 0.14 | 3.16 | 4.29 |
| *Ziziphus mauritiana* | 0.99 | 0.25 | 2.94 | 4.18 |
| *Ficus sycomorus* | 0.99 | 0.22 | 2.93 | 4.14 |
| *Lannea fructicosa* | 0.99 | 0.39 | 2.75 | 4.12 |
| *Celtis integrifolia* | 0.99 | 0.22 | 2.89 | 4.10 |
| *Feretia apodanthera* | 2.46 | 1.44 | 0.04 | 3.95 |
| *Ximenia americana* | 2.46 | 0.03 | 1.18 | 2.19 |
| *Ficus ingens* | 0.49 | 0.89 | 0.49 | 3.84 |
| *Combretum fragrans* | 1.48 | 0.08 | 3.22 | 3.79 |
| *Lannea velutina* | 0.49 | 0.72 | 1.55 | 3.75 |
| *Azadirachta indica* | 1.48 | 0.36 | 2.76 | 3.62 |
| *Maytenus senegalensis* | 1.48 | 0.19 | 1.78 | 3.45 |
| *Ziziphus mucronata* | 1.97 | 0.47 | 1.38 | 3.33 |
| *Bauhinia rufescens* | 1.97 | 0.47 | 0.85 | 3.29 |
| *Piliostigma thonningii* | 1.48 | 0.67 | 0.63 | 3.27 |
| *Dichrostachys cinerea* | 1.97 | 0.42 | 1.35 | 3.25 |
| *Capparis sepiaria* | 1.97 | 0.67 | 0.44 | 3.08 |
| *Ziziphus spina-christii* | 1.48 | 0.83 | 0.05 | 2.86 |
| *Gardenia ternifolia* | 1.48 | 0.17 | 1.15 | 2.79 |
| *Hyphaene thebaica* | 0.99 | 0.42 | 0.77 | 2.66 |
| *Prosopis africana* | 0.99 | 0.14 | 1.40 | 2.52 |
| *Flueggea virosa* | 1.48 | 0.31 | 1.18 | 2.47 |
| *Dalbergia melanoxylon* | 1.48 | 0.19 | 0.73 | 2.40 |
| *Ficus gnaphalocarpa* | 0.49 | 0.42 | 0.49 | 2.39 |
| *Strychnos spinosa* | 0.99 | 0.19 | 1.61 | 2.30 |
| *Pterocarpus erinaceus* | 0.49 | 0.22 | 1.04 | 2.25 |
| *Combretum molle* | 0.99 | 0.25 | 1.45 | 2.19 |
| *Senegalia polyacantha* | 1.48 | 0.25 | 0.86 | 2.10 |
| *Terminalia avicennioides* | 0.49 | 0.19 | 0.11 | 1.78 |
| *Cadaba farinosa* | 0.99 | 0.39 | 0.77 | 1.65 |
| *Grewia flavescens* | 0.99 | 0.44 | 0.01 | 1.44 |
| *Swartzia madagascariensis* | 0.99 | 0.17 | 0.05 | 1.21 |
| *Vitex doniana* | 0.49 | 0.19 | 0.01 | 1.19 |
| *Grewia villosa* | 0.99 | 0.03 | 0.67 | 1.19 |
| *Parkia biglobosa* | 0.99 | 0.14 | 0.05 | 1.18 |
| *Calotropis procera* | 0.99 | 0.08 | 0.05 | 1.12 |
| *Terminalia macroptera* | 0.99 | 0.08 | 0.01 | 1.08 |
| *Combretum aculeatum* | 0.49 | 0.36 | 0.04 | 0.89 |
| *Capparis corimbosa* | 0.49 | 0.17 | 0.05 | 0.71 |
| *Albizia lebbeck* | 0.49 | 0.03 | 0.04 | 0.56 |

Supplementary Table S3: List of medicinal plants including the parts used, methods of preparation, route of administration, and treated diseases

| Medicinal species | Plant parts used | Ailments treated | Preparation methods | Modes of administration |
| --- | --- | --- | --- | --- |
| *Faidherbia albida* | Bark | Flu et cough | Decoction | Oral |
| *Vachellia nilotica* | Leaves, fruit, Bark | Hemorrhoid, stomach ache | Decoction, infusion | Oral |
| *Vachellia seyal* | Bark | Stomach ache | Decoction | Oral |
| *Annona senegalensis* | Fruits, bark, root | Diarrhea, snakebite, sterility | Decoction | Oral |
| *Terminalia leiocarpa* | Bark, root | Fever yellow | Infusion | Oral |
| *Azadirachta indica* | Leaves, fruits | Stomach ache, malaria | Decoction, oil extraction | Oral |
| *Balanites aegyptiaca* | Fruits | Stomach ache, breast milk problem | Maceration | Oral |
| *Bauhinia rufescens* | Leaves, wood | Typhoid | Decoction | Oral |
| *Bombax costatum* | Bark, root | Diarrhea, fever | Decoction | Oral |
| *Calotropis procera* | Bark, root | Scabies, itching | Decoction | Oral , cutaneous |
| *Combretum aculeatum* | Bark, root | Syphilis, digestive disorders | Decoction | Oral |
| *Combretum collinum* | Bark, root | Wounds, gonorrhoea | Decoction | Oral, cutaneous |
| *Combretum fragrans* | Bark, root | Fever, malaria, diarrhea | Decoction, infusion | Oral |
| *Dalbergia melanoxylon* | Bark, root | Headaches, malaria | Decoction | Oral |
| *Diospyros mespiliformis* | | Hemorrhage, cough, diarrhea | Decoction | Oral |
| *Feretia apodanthera* | Leaves, bark, root | Malaria, dysentery | Decoction | Oral |
| *Ficus sycomorus* | Root | Whooping cough | Decoction | Oral |
| *Guiera senegalensis* | Bark, root | Wounds, inflammation, conjunctivitis | Decoction | Oral, cutaneous |
| *Kaya senegalensis* | Bark, root, seed | Malaria, fever, stomach ache | Decoction, oil extraction | Oral, pomade |
| *Kigelia americana* | Fruit, bark | Infertility, dysentery | Decoction | Oral |
| *Maytenus senegalensis* | Leaves, bark | Gonorrhea, toothache | Decoction | Oral |
| *Piliostigma reticulatum* | leaves, bark, root | Measles, diarrhea | Decoction | Oral |
| *Prosopis africana* | Leaves, bark, root | Ulcer, rheumatism | Decoction | Oral, cutaneous |
| *Pseudocedrela kotschyi* | Bark, root | Fever, malaria, ulcer | Decoction | Oral |
| *Pterocarpus erinaceus* | Bark, root | Anemia, sterlity, ulcer, wound | Decoction, oil extraction | Oral, pomade |
| *Sclerocarya birrea* | Bark, root | Toothache, diarrhea | Decoction | Oral |
| *Steriospermum kunthianum* | Bark, root | Bilharzia, syphilis, gonorrhea, cough diarrhea | Decoction, maceration | Oral , cutaneous |
| *Strychnos spinosa* | Leaves, bark, root | Gastrite, gonorrhoea | Decoction, infusion | Oral, cutaneous |
| *Swartzia madagascariensis* | Bark, root | Malaria, fever yellow, hepatitis | Maceration | Oral |
| *Tamarindus indica* | Fruit, leave, root | Malaria, varicella ,cough | Maceration, decoction | Oral, cutaneous |
| *Terminalia macroptera* | Bark, root | Syphilis, gonorrhea, malaria, diarrhea | Decoction, infusion | Oral, sream bath |
| *Ximenia americana* | Bark, root | Wounds, sexual impotence, cough, gonorrhoea | Infusion | Oral |
| *Ziziphus mauritiana* | Bark, root | Stomach ache | Decoction | Oral |
| *Ziziphus mucronata* | Bark, root | Ulcer, fever | Decoction | Oral, sream bath |

Supplementary Table S4: Citation frequencies of food species in the two forest massifs

|  | Citation frequency (%) | | |
| --- | --- | --- | --- |
| Food species | Plant parts used | Magayel forest | Barkaya forest |
| *Crateva adansonii* | Leaves | 2.27 | - |
| *Annona senegalensis* | Fruits | 13.91 | 23.37 |
| *Balanites aegyptiaca* | Leaves, flowers, fruits, seeds | 40.54 | 40.63 |
| *Capparis sepiaria* | Fruits | 4.15 | 1.25 |
| *Celtis integrifolia* | Fruits, leaves | 1.06 | 1.25 |
| *Adansonia digitate* | Fruits, leaves | 2.27 | - |
| *Diospyros mespilisformis* | Fruits | 3.34 | - |
| *Ficus gnaphalocarpa* | Fruits | 27.22 | 25.60 |
| *Ficus ingens* | Fruits | 12.89 | 18.79 |
| *Ficus platyphylla* | Fruits | 9.87 | 8.06 |
| *Ficus sycomorus* | Fruits | 14.22 | 9.03 |
| *Bombax costatum* | Flowers | 10.97 | 11.57 |
| *Gardenia ternifolia* | Fruits | 4.35 | 6.81 |
| *Grewia villosa* | Fruits | 1.06 | - |
| *Hexalobus monopetalus* | Fruits | 40.43 | 41.43 |
| *Hyphaene thebaica* | Fruits | 34.76 | 35.12 |
| *Piliostigma thonningii* | Fruits | 12.11 | 15.04 |
| *Sclerocarya birrea* | Fruits, seeds | 21.45 | 11.81 |
| *Strychnos spinosa* | Fruits | 4.09 | 3.47 |
| *Tamarindus indica* | Leaves, flowers, fruits | 45.59 | 46.69 |
| *Vitex doniana* | Fruits | 22.82 | 17.54 |
| *Ximenia Americana* | Fruits | 16.24 | 16.39 |
| *Ziziphus mauritiana* | Fruits | 33.70 | 41.65 |
| *Ziziphus spina-christii* | Fruits | 20.69 | 24.52 |

Supplementary Table S5: Citation frequencies of medicinal species in the two forest massifs

| Citation frequency (%) | | | |
| --- | --- | --- | --- |
| Medicinal species | Plant parts used | Magayel forest | Barkaya forest |
| *Faidherbia albida* | Bark | 22.82 | 26.88 |
| *Vachellia nilotica* | Leaves, fruit, bark | 45.00 | 49.01 |
| *Vachellia seyal* | Bark | 2.07 | 1.25 |
| *Annona senegalensis* | Fruit, bark, root | 6.59 | 7.08 |
| *Terminalia leiocarpa* | Bark, root | 52.68 | 10.04 |
| *Azadirachta indica* | Leaves, fruit | 23.07 | 65.06 |
| *Balanites aegyptiaca* | Fruit | 38.30 | 21.85 |
| *Bauhinia rufescens* | Leaves, bois | 4.15 | 46.23 |
| *Bombax costatum* | Bark, root | 17.50 | 2.50 |
| *Calotropis procera* | Bark, root | 2.07 | 21.85 |
| *Combretum aculeatum* | Bark, root | 2.07 | 1.25 |
| *Combretum collinum* | Bark, root | 2.07 | 1.25 |
| *Combretum fragrans* | Bark, root | 8.49 | 1.25 |
| *Dalbergia melanoxylon* | Bark, root | 4.15 | 3.47 |
| *Diospyros mespilisformis* | Bark, root, leaves | 2.07 | 2.50 |
| *Feretia apodanthera* | Leaves, bark, root | 4.15 | 1.25 |
| *Ficus sycomorus* | Root | 8.39 | 2.50 |
| *Guiera senegalensis* | Bark, root | 3.08 | 1.25 |
| *Kaya senegalensis* | Bark, root, seed | 52.46 | 8.23 |
| *Kigelia americana* | Fruit, bark | 2.07 | 1.25 |
| *Maytenus senegalensis* | Leaves, bark | 7.29 | 48.41 |
| *Piliostigma reticulatum* | Leaves, bark, root | 6.42 | 1.25 |
| *Prosopis africana* | Leave, bark, root | 1.06 | 1.25 |
| *Pseudocedrela kotschyi* | Bark, root | 3.08 | 5.00 |
| *Pterocarpus erinaceus* | Bark, root | 3.08 | 4.72 |
| *Sclerocarya birrea* | Bark, root | 3.34 | 1.25 |
| *Steriospermum kunthianum* | Bark, root | 2.07 | 3.47 |
| *Strychnos spinosa* | Leaves, bark, root | 2.27 | 1.25 |
| *Swartzia madagascariensis* | Bark, root | 26.41 | 5.56 |
| *Tamarindus indica* | Fruit, leave, root | 5.36 | 23.23 |
| *Terminalia macroptera* | Bark, root | 2.27 | 2.22 |
| *Ximenia americana* | Bark, root | 4.09 | 1.25 |
| *Ziziphus mauritiana* | Bark, root | 27.90 | 23.93 |
| *Ziziphus mucronata* | Bark, root | 2.07 | 1.25 |

Supplementary Table S6: Citation frequencies of fodder species in the two forest massifs

| Fodder species | Citation frequency (%) | | |
| --- | --- | --- | --- |
|  | Plant parts used | Magayel forest | Barkaya forest |
| *Faidherbia albida* | Leaves, fruits | 33.20 | 18.37 |
| *Vachellia seyal* | Leaves | 20.04 | 21.85 |
| *Vachellia sieberiana* | Leaves | 24.39 | 3.47 |
| *Terminalia leiocarpa* | Leaves | 9.86 | 12.82 |
| *Balanites aegyptiaca* | Leaves, fruits | 15.39 | 4.72 |
| *Bombax costatum* | Leaves, flowers | 9.16 | 8.06 |
| *Dichrostachys cinerea* | Leaves | 9.86 | 5.97 |
| *Ficus ingens* | Leaves fruits | 13.31 | 12.82 |
| *Ficus platyphylla* | Leaves, fruits | 33.25 | 39.42 |
| *Ficus sycomorus* | Leaves, fruits | 37.91 | 38.97 |
| *Ficus thonningii* | Leaves, fruits | 13.51 | 15.04 |
| *Hexalobus monopetalus* | Leaves | 9.16 | 6.81 |
| *Piliostigma thonningii* | Leaves | 5.52 | 12.82 |
| *Prosopis africana* | Leaves | 11.24 | 24.38 |
| *Sclerocarya birrea* | Leaves, fruits | 21.11 | 41.72 |
| *Sterculia setigera* | Leaves | 42.20 | 21.01 |
| *Steriospermum kunthianum* | Leaves | 10.17 | 44.01 |
| *Tamarindus indica* | Leaves, flowers, fruits | 22.01 | 29.11 |
| *Ziziphus mauritiana* | Leaves, fruits | 33.25 | 34.62 |
| *Ziziphus spina-christii* | Leaves, fruits | 25.45 | 4.72 |

Supplementary Table S7: Citation frequencies of firewood species in the two forest massifs

| Firewood species | Citation frequency (%) | | |
| --- | --- | --- | --- |
|  | Plant parts used | Magayel forest | Barkaya forest |
| *Faidherbia albida* | Wood | 17.66 | 14.86 |
| *Senegalia ataxacantha* | Wood | 2.07 | 1.25 |
| *Vachellia nilotica* | Wood | 2.07 | 1.25 |
| *Senegalia polyacantha* | Wood | 2.07 | - |
| *Vachellia seyal* | Wood | 18.36 | 19.35 |
| *Vachellia sieberiana* | Wood | 15.23 | 18.37 |
| *Andira inermis* | Wood | 7.79 | 11.57 |
| *Annona senegalensis* | Wood | 2.07 | 1.25 |
| *Terminalia leiocarpa* | Wood | 35.16 | 36.88 |
| *Azadirachta indica* | Wood | 13.35 | 15.04 |
| *Balanites aegyptiaca* | Wood | 15.17 | 32.62 |
| *Bombax costatum* | Wood | 2.07 | 1.25 |
| *Calotropis procera* | Wood | 2.07 | 1.25 |
| *Capparis sepiaria* | Wood | 2.07 | 1.25 |
| *Celtis integrifolia* | Wood | 7.79 | 11.57 |
| *Combretum aculeatum* | Wood | 5.72 | 10.32 |
| *Combretum collinum* | Wood | 4.15 | 2.50 |
| *Combretum fragrans* | Wood | 11.94 | 9.31 |
| *Combretum glutinosum* | Wood | 11.94 | 12.82 |
| *Combretum molle* | Wood | - | 2.50 |
| *Crateva adansonii* | Wood | - | 2.22 |
| *Dalbergia melanoxylon* | Wood | 2.07 | 1.25 |
| *Dichrostachys cinerea* | Wood | 4.15 | 2.50 |
| *Diospyros mespilisformis* | Wood | 14.02 | 14.07 |
| *Feretia apodanthera* | Wood | 2.07 | - |
| *Ficus gnaphalocarpa* | Wood | 2.07 | 1.25 |
| *Ficus ingens* | Wood | 14.22 | 9.03 |
| *Ficus platyphylla* | Wood | 12.14 | 15.04 |
| *Ficus sycomorus* | Wood | 4.15 | 2.50 |
| *Ficus thonningii* | Wood | 9.87 | 12.82 |
| *Gardenia ternifolia* | Wood | 2.07 | 1.25 |
| *Guiera senegalensis* | Wood | 4.35 | 3.47 |
| *Hexalobus monopetalus* | Wood | 2.07 | 1.25 |
| *Hyphaene thebaica* | Wood | 4.35 | 4.72 |
| *Kigelia americana* | Wood | 2.07 | - |
| *Maytenus senegalensis* | Wood | 2.07 | 1.25 |
| *Piliostigma reticulatum* | Wood | 19.74 | 16.29 |
| *Piliostigma thonningii* | Wood | 11.94 | 12.82 |
| *Prosopis africana* | Wood | 30.50 | 31.33 |
| *Pseudocedrela kotschyi* | Wood | 23.07 | 16.29 |
| *Pterocarpus erinaceus* | Wood | 4.15 | 2.50 |
| *Sclerocarya birrea* | Wood | 7.79 | 11.57 |
| *Senna singueana* | Wood | 2.07 | 1.25 |
| *Steriospermum kunthianum* | Wood | 12.14 | 10.28 |
| *Strychnos spinosa* | Wood | 2.07 | 1.25 |
| *Swartzia madagascariensis* | Wood | 4.15 | 1.25 |
| *Tamarindus indica* | Wood | 4.15 | 2.50 |
| *Terminalia avicennioides* | Wood | 1.25 | - |
| *Terminalia macroptera* | Wood | 2.07 | 1.25 |
| *Vitex doniana* | Wood | 2.07 | 1.25 |
| *Ximenia americana* | Wood | 5.72 | - |
| *Ziziphus mauritiana* | Wood | 2.07 | 1.25 |
| *Ziziphus mucronata* | Wood | 7.79 | 6.81 |
| *Ziziphus spina-christii* | Wood | 2.07 | 1.25 |
